# Coevolution of Quantum and Classical Strategies on Evolving Random Networks

**DOI:** 10.1371/journal.pone.0068423

**Published:** 2013-07-12

**Authors:** Qiang Li, Azhar Iqbal, Matjaž Perc, Minyou Chen, Derek Abbott

**Affiliations:** 1 State Key Laboratory of Power Transmission Equipment and System Security and New Technology, College of Electrical Engineering, Chongqing University, Chongqing, China; 2 School of Electrical and Electronic Engineering, University of Adelaide, Adelaide, Australia; 3 Department of Physics, Faculty of Natural Sciences and Mathematics, University of Maribor, Maribor, Slovenia; 4 Department of Mathematics and Statistics, King Fahd University of Petroleum and Minerals, Dhahran, Kingdom of Saudi Arabia; Centre de Physique Théorique, France

## Abstract

We study the coevolution of quantum and classical strategies on weighted and directed random networks in the realm of the prisoner’s dilemma game. During the evolution, agents can break and rewire their links with the aim of maximizing payoffs, and they can also adjust the weights to indicate preferences, either positive or negative, towards their neighbors. The network structure itself is thus also subject to evolution. Importantly, the directionality of links does not affect the accumulation of payoffs nor the strategy transfers, but serves only to designate the owner of each particular link and with it the right to adjust the link as needed. We show that quantum strategies outperform classical strategies, and that the critical temptation to defect at which cooperative behavior can be maintained rises, if the network structure is updated frequently. Punishing neighbors by reducing the weights of their links also plays an important role in maintaining cooperation under adverse conditions. We find that the self-organization of the initially random network structure, driven by the evolutionary competition between quantum and classical strategies, leads to the spontaneous emergence of small average path length and a large clustering coefficient.

## Introduction

Evolutionary games on graphs and networks as well as coevolutionary games have recently received significant attention [1]–[4]. Nowak and May’s discovery of network reciprocity [5] has indeed spawned a spree of activity aimed at understanding how the interactions between us affect the evolution of cooperation. The later has implications ranging from the Cold War to bacterial colonies [6], [7]. While there are other forms of reciprocity that one can count on to lead to cooperation [8], network reciprocity has received a substantial push from leaps of progress in network science that have unfolded roughly a decade ago [9]–[12]. Evolutionary games have been staged on various types of complex networks [13]–[27], whereby in particular the scale-free network has been identified as an excellent host topology for cooperative individuals [28]–[30], warranting the best protection against defectors. Since the strong heterogeneity of the degree distribution of scale-free networks was identified as a key driving force behind flourishing cooperative states [31]–[35], some alternative sources of heterogeneity were also investigated as potential promoters of cooperation with noticeable success (see also [36]). Examples of such approaches include the introduction of preferential selection [37], asymmetry of connections [38], different teaching capabilities [39], heterogeneous influences [40], social diversity [41] as well as diversity of reproduction time scales [42]. Coevolutionary games [3] have also been extensively studied, for example in the study of the coevolution of strategy and structure [43], games on networks subject to random or intentional rewiring procedures [14], [44]–[51], prompt reactions to adverse ties [52], [53], games on growing networks [54], [55], and indeed many more [49], [56]–[65], [65,66].

While classical game theory [67]–[69] has made an impact on a large range of disciplines, it has also been generalized to the quantum regime [73], [74]. A new research area dubbed *quantum game theory* has emerged, and has since attracted considerable attention. Some interesting results without counterparts in classical game theory have been reported. For example, an agent using a quantum strategy can always defeat an opponent using a classical strategy and increase expected payoffs in a penny flip game [74]. When the Prisoner’s Dilemma (PD) is quantized, it is surprising that the dilemma in the PD can be escaped if agents are allowed to play quantum strategies in a restricted space [73]. Later, the Battle of the Sexes game was studied in a further quantum game model, and a unique equilibrium for the game was found, provided agents adopt quantum strategies [75]. Furthermore, the model for a two-person quantum game has been extended to a 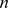-person quantum game [76]. Later on, evolutionary quantum games [76], evolutionary stable strategies [77], quantum cooperative games [78] and quantum repeated games [79] were also studied. More recently, a unifying perspective on both the classical and quantum versions of two-player games has been given by a probabilistic framework [80]. Classically defined games have been analyzed and it has been found that a quantum team has an advantage over any classical team [81]. Quantum games have also been analyzed by using geometric algebra [82]–[84], and they have been implemented using quantum computers [85]–[88]. For further background on quantum games, we refer to [89], [90].

It is important to note that quantum games are established on quantum mechanics, and hence quantum effects such as entanglement can be employed, which may give rise to results or phenomena without classical counterparts. This is also the main difference between a quantum and a classical game. According to quantum game theory, a classical strategy set is only a subset of the full quantum strategy space, and the latter can thus be used to describe a larger variety of different phenomena. If agents can use both quantum and classical strategies, an interesting question is how these strategies evolve on a network. Previous research [70]–[72] has shown that the evolution exhibits new features without classical counterparts. For example, if strategies evolve on a static network, a quantum strategy becomes the dominant strategy in the population from the outset, when a PD game is employed. Conversely, if only the two classical strategies of cooperation (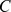) and defection (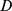) are considered, defectors always dominate for sufficiently large temptations to defect. In Ref [72], the evolution of quantum and classical strategies was studied in spatial public goods games, where cooperators could survive even at 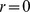, while in the classical regime there exists at critical finite 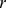 where cooperators die out. These results, however, were obtained on *static* networks.

In this paper, we therefore focus on the behavior of quantum and classical strategies on an *evolving* network, where relationships among agents vary over time by means of a coevolutionary process. As is observed in societies, friendship networks consist of a set of relationships weighted by the level of trust between friends. Trust can increase or decrease depending on the past actions of each member of the network. The process of making friends can be modeled by a weighted and directed evolving network, where agents are regarded as nodes of the network, relationships between them as links and the degree of trust as a weight on a link. Therefore, in this paper, the evolution of quantum and classical strategies on a weighted and directed evolving network is investigated. In the evolving network, the structure of the network varies with time due to agents switching their neighbors, which is implemented by breaking links and connecting new ones. Further, if there are two directed links between two nodes, this means that two agents (the nodes) are best friends and the degrees of trust (the weights on the links) are highest. When a link is broken and rewired to a new node, it means an agent makes a new friend, and then the new friend assigns a degree of trust (a weight) to the relationship (the link). Over time, the degree of trust or the weight on a given link can increase or decrease by agents breaking and rewiring links that belong to them. It is worth noting that an agent cannot cut the links directed from its neighbors to itself, but it can lower the weights on these links to punish the neighbors. Also, it should be emphasized that the direction of a link only indicates to whom the link belongs, but two agents are neighbors if there is a link between them, regardless of the direction of the link, and they can adopt strategies from one another likewise unrestricted by the directionality of links.

The evolution of the network and the modification of weights can be visualized in terms of a game-theoretic setting with associated payoffs, i.e., links and weights are altered as a function of the set of payoffs, and meanwhile agents’ total payoffs are affected by weights too. Furthermore, an agent’s total payoff will influence the spread of a strategy in the network. Obviously, high total payoffs are advantageous to the wide spread of strategies. Based on the rules of coevolution, new patterns are observed, when quantum games and quantum strategies are involved. Further, we discuss the coevolution in different parameters and explain the results of the evolution of strategies and networks in detail. It is worth noting that a quantum strategy is not a probabilistic sum of pure classical strategies (except under special conditions), and that it cannot be reduced to pure classical strategies [77].

The basics of quantum games and the model with coevolutionary rules are presented in the Methods section, where also the notation and other mathematical concepts are introduced. Next we proceed with the results, in particular showing how the probability of a structural update event influences the evolution of quantum and classical strategies, what is the impact of the relationship between the coevolution and the number of initial neighbors, and what is the impact of minimizing weights. Lastly we also investigate the statistical properties of the interaction networks before and after the coevolution. We conclude with a brief discussion of presented results.

## Methods

### Basics of Quantum Games

The Prisoner’s Dilemma, as an abstraction of many strategic phenomena in the real world, has been widely applied in a number of scientific fields. In this symmetric game, each agent has two available strategies, Cooperation (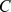) and Defection (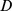). If both agents are cooperators, then they receive Reward (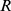). Contrarily, if they are both defectors, they receive Punishment (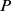). When one is a cooperator and the other is a defector, the cooperator receives Sucker (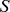), while the defector acquires the highest payoff, Temptation (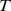). So, the payoff matrix to the focal agent can be written as
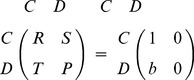
(1)


To be compatible with previous studies and without loss of generality, the payoff matrix of the PD game is chosen as 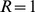, 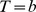 (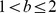), 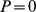 and 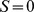, satisfying the inequalities 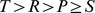. As is known in classical game theory, the strategy profile 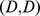 is the unique Nash Equilibrium (NE). However, the strategy profile 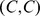 is merely the best choice that is Pareto optimal [91]. This gives rise to the dilemma.

**Figure 1:**
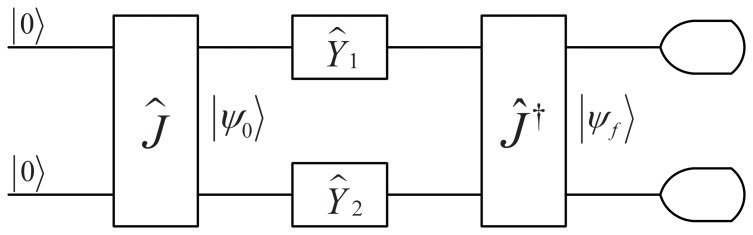
A model of a quantum game. For details on the notation we refer to the Methods section, in particular the subsection Basics of Quantum Games.

On the other hand, if both agents are allowed to adopt quantum strategies in a restricted space, the dilemma can be removed [73]. Next, we will introduce the model of a quantum game briefly, which is shown in Fig. 1 [73].

In the model, at first two basis vectors 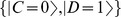 in Hilbert space are assigned to the possible outcomes of the classical strategies, 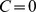 and 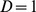, respectively [73]. Assume a quantum game starts in an initial state 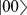, where two qubits belong to two agents, say Alice and Bob. The state will be 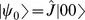, if the initial state is operated by a unitary operator 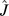 that is known to both agents. For a 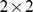 maximally entangled quantum game, the entangling operator 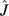 takes form below [92], [93]

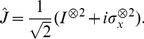
(2)


In the following, each agent chooses a unitary operator 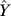 as a strategy from the full quantum unitary strategy space 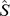 [89],
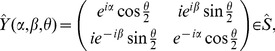
(3)where 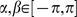, 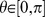 and then operates it on the qubit that belongs to the agent. Finally, the state 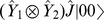 goes through a unitary operator 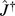. Before Alice and Bob forward their qubits for the final measurement, i.e., before a projective measurement on the basis 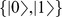 is carried out, the final state is



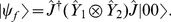
(4)Thus, the focal agent’s expected payoff can be calculated as
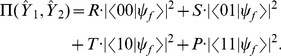
(5)


### The Model with Coevolutionary Rules

Assume there is a weighted and directed network 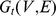 with 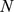 nodes, where 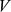 is the set of nodes, 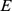 is the set of links and 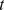 is the time step. There are no duplicated links and self loops in the network. Initially, a regular random network 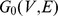 is constructed, in which each node has 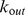 neighbors, which warrants that all nodes have equal chances of success [94]. Here, 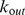 is the outdegree of a node. Moreover, initially there are two links between any pairs of connected nodes and the initial weight on each link is 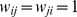. A regular random network can be created as follows. At first, a undirected ring with 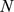 nodes is constructed, where each node has 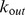 nearest neighbors. Next, we choose two links randomly in the ring, say {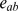 and 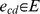}, and switch two nodes belonging to different links to created two new links, {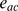 and 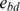} or {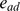 and 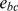}. Then, we check if the number of neighbors of each node is 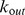 or not. If the numbers of neighbors of all nodes are 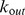, then the two new links will be retained. Otherwise, the switch operation will be canceled. The two steps are repeated till all links in the network are rewired once.

Each node 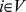 in the network is occupied by an agent and its neighbor 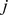 is any other agent such that there is a link between them, so the set of neighbors of an agent 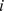 at a time step 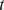 can be defined as
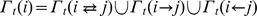


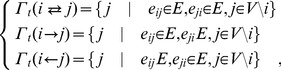
(6)


where 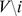 means the set of nodes, 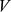, not including the 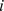-th node (a complement of 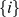 in 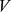) and there are two links between the agent 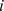 and its neighbor 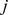 in 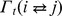, also called *bidirectional links*. Similarly, there is a directed link from the agent 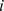 to the neighbor 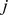 in 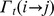, while there is a link directed from the neighbor 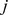 in 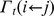 to the agent 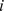. According to the definition, any two agents are neighbors, only if there is a link between them, regardless of the direction of the link. And the total number of neighbors of the agent 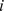 is 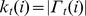, where 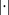 represents the cardinality of a set.

Initially, each agent on the network is randomly assigned one of two quantum strategies or two classical strategies (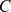 and 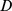) with equal probability, all of which are taken from the full quantum strategy space 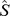, and the initial fraction of agents using each strategy is equal. Particularly, the classical strategies, 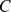 and 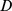, take the forms:
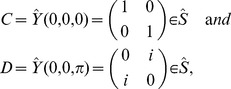
(7)while two quantum strategies, 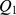 and 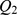, are produced by choosing the parameters, 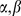 and 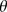, in Eq. 3 randomly, before each simulation starts. For example, at the 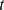-th simulation, initially, 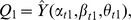 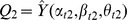.

Next, the rules of the strategy evolution and the network evolution are introduced in detail. *Strategy evolution*: (i) a randomly selected agent 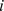 plays 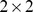 maximally entangled quantum games with all its neighbors in 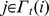, respectively, according to the model of a quantum game (Fig. 1). The expected payoff of the agent after playing a game with a neighbor can be calculated by Eq. 5, 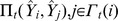, while its total payoff 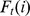 is written as
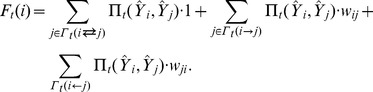
(8)(ii) After each round, the agent 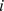 randomly chooses a neighbor 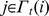, and then the agent 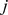 calculates its total payoff 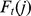 in terms of the above mentioned method. In the framework of the replicator dynamics, the agent 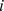 compares its total payoff with the neighbor’s and imitates the neighbor’s strategy with probability 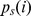, which is given as
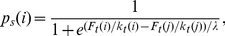
(9)where 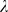 is the intensity of selection and the updating rule is also called the Fermi rule. If the agent 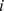 decides to imitate this strategy, it will play it in the next round. It is worth noting that the direction of a link between two agents only represents who controls this link and agents can adopt strategies from one another likewise unrestricted by the directionality of links. This process is called a strategy update event.

**Figure 2:**
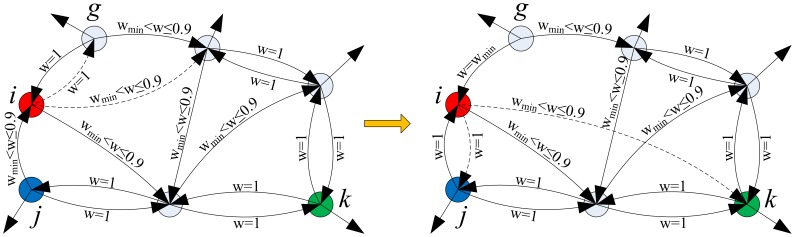
Illustration of the network evolution. The left panel represents the status of the network before the network evolution, in which the focal agent 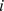 (in red) on the network with 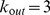 intends to break two links (in dash lines) due to payoffs less than the average. The right panel is the status of the network after the network evolution, where the focal agent preferentially rewires one of the broken links to a neighbor 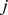 (in blue) who brings a payoff greater than the average and upgrades the weights to 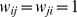. However, the weight on the remaining link between the agent 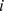 and 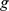 is set at 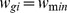 by the agent 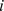 for punishment. Then, it chooses a neighbor’s neighbor 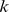 (in green) at random and rewires the other link to the neighbor 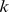. Meanwhile, the neighbor 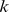 assigns a weight 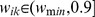 randomly to the new link.

*Network evolution*: After the agent updates its strategy, the structure of the network is updated with probability 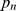. First, agent 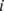 identifies the neighbors who bring payoffs that are below the average and those with minimal weights on links, and then puts them in a set 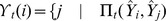 < 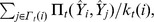 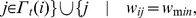 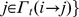, where 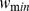 represents the minimal weight. In the set, there exist three types of links between the agent and the neighbors: (i) bidirectional links, 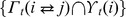; (ii) links directed from the agent to the neighbors, 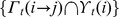; (iii) links directed from the neighbors to the agent, 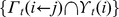. For Case (i), the agent performs the following three steps. (a) Link broken. The agent breaks the links that belong to it. (b) Link rewired. The broken links are preferentially rewired to the neighbors who bring payoffs higher than the average and where there is only one link directed from each of the neighbors to the agent, 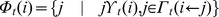. As such, there are two links between the agent and a neighbor in 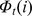, and the weights on the links are upgraded to 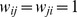. If the number of broken links is larger than that of 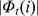, the other links will be rewired to the agent’s neighbors’ neighbors at random [94], satisfying the condition that there are no links between the chosen nodes and the agent before rewiring. The new neighbors will randomly assign weights 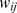 to the new links, which are restricted to an interval 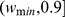, and follow a normal distribution with 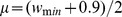 and 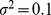, where 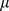 and 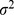 are the mean and the variance, respectively. This distribution is applied in order to imitate that most people in reality give half degrees of trust to new friends, when 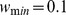. (c) Punishment. The weights on the links directed from the neighbors to the agent are set to 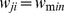 for punishment. In Case (i), these links remain after Step (a), because the agent only breaks the links directed from it to the neighbors. On the other hand, for Case (ii), the agent only needs to do Step (a) and (b), because all links are directed from the agent to neighbors. It is easier for Case (iii), because only Step (c) needs to be carried out. The process of the network evolution is illustrated in Fig. 2, which is also called a *structural update event*.

The entire game is iterated for a maximum number of 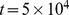 time steps and the fractions of agents with different strategies are calculated by averaging over another 1000 time steps after the maximum, which produces a result of evolution of strategies 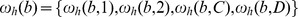, where 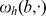 denotes the fraction of agents with a certain strategy at a given 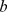. When the temptation 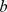 changes from 1 to 2, 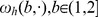 represents a curve and 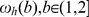 represents a family of curves. The statistical result 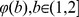 is obtained by averaging over at least 200 of these results 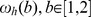, namely, 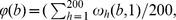 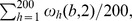 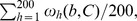 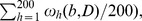 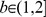. If strategies of all agents do not change for 1000 consecutive time steps, it is deemed that a steady state has been reached and the iteration ends.

## Results and Discussion

In our simulations, the coevolution starts from a weighted and directed regular random network 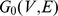 with 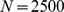 nodes that are occupied by agents using quantum and classical strategies. Agents play games with their immediate neighbors according to the model of a quantum game. Due to the rules of the coevolution involved, agents can break and rewire their own links, which leads the network to become an evolving network 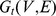. During the coevolution, the intensity of selection is set at 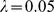 throughout the paper and the weight for punishment is set at 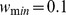, if not otherwise explicitly stated. Later, the coevolution of strategies and networks over different parameters is investigated.

**Figure 3:**
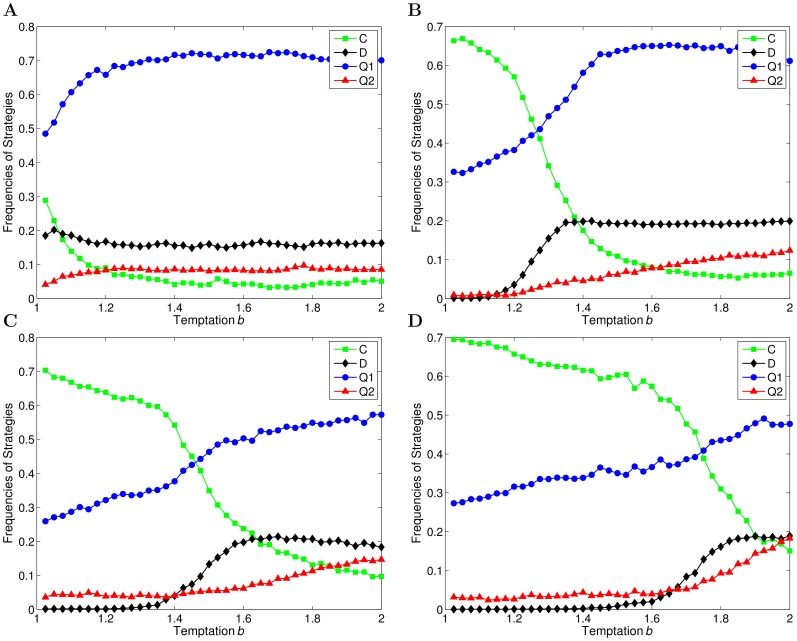
The evolution of strategies as a function of 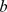 on a weighted and directed evolving network for different 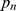. (a) 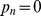. (b) 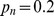. (c) 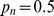. (d) 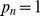. (a)–(d) exhibit the fractions of agents using two quantum strategies (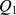 and 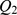) and two classical strategies (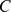 and 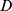) in the population after the coevolution, when 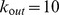, 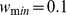 and different probabilities of a structural update event are adopted.

In this section, how the probability of a structural update event occurring influences the evolution of quantum and classical strategies is studied first, and then the results are explained in detail. Fig. 3 exhibits the statistical results of the evolution of four strategies on an evolving network with different probabilities 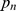. Because quantum strategies are taken from a very large space 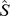 by choosing the parameters, 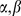 and 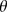 at random, before each simulation starts, the final result 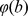 is obtained statistically in order to reduce randomness. In the result of each simulation 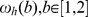 (like Fig. 3), for the curves corresponding to the quantum strategies, the quantum strategy that produces the topmost curve is defined as 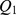, the second curve as 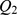, and so on. Finally, the statistical result 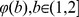 can be obtained in terms of the statistical method described in the last of the Methods section.

In Fig. 3, there exists a curve that is higher than others, when the coevolution comes to an end, which means that the strategy is played by most of agents in the population and it is also a dominant strategy in the population. When the strategies evolve on a static network, namely 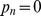, a quantum strategy can be a dominant strategy from the outset. Furthermore, the fraction of agents using the dominant strategy rises slightly with the increase of the temptation 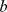. However, once the network evolution is involved, new patterns emerge in the evolution of strategies. As is shown in Fig. 3 (b)–(d), even if the probability of a structural update event 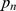 is low, the strategy, Cooperation, dominates in the population when 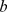 is small. However, as 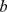 increases, the fractions of agents using quantum strategies in the population exceed that of cooperators gradually, and finally a quantum strategy becomes the dominant strategy. Moreover, the fraction of defectors also increases a little at the same time.

When an agent adopts a quantum strategy 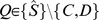 against its neighbor who uses a strategy 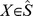, according to Eq. 5, its expected payoff is restricted to an interval 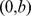, i.e., 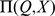 or 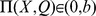. Further, based on the statistical analysis of payoffs, most payoffs of agents using the strategy 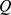 are less than 1, 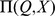 or 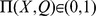, when 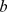 is less than the critical value. In terms of the rules of the network evolution, if the focal agent’s payoff, after a game with a neighbor, is less than the average, the link directed from the focal agent to the neighbor will be broken. In order to observe the behavior of different pairs of agents, we list the focal agent’s payoffs and possible operations in Table 1 according to the statistical analysis, before 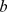 reaches the critical value. Here, a 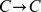 pair means two agents connected by a link both adopt the strategy 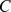, a 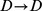 pair means both adopt the strategy 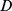, and so on. From Table 1, it can be found that 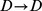 and 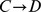 pairs are surely broken, because the focal agent’s payoff is zero, and other pairs will be broken if the received payoffs are less than the average or the weights of the links are 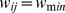. On the contrary, 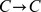 pairs always bring 1 for each agent, while the payoff 1 is greater than the average easily at a small 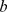, so that many 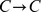 pairs can be preserved. Furthermore, when rewiring begins, it is more likely that agents preferentially rewire the broken links to the cooperators in the neighborhood, because they bring payoffs greater than the average. As such, the number of 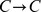 pairs will be increased further. Hence, cooperators can accumulate higher payoffs and the strategy 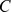 can spread widely in the population when 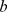 is small.

**Table 1:** The focal agents’ possible operations on different pairs according to the statistical analysis of payoffs, before the critical value of 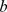.

Pairs	Payoffs	Operations
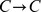	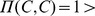 Average	Preserved
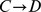	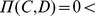 Average	Broken and Rewired
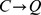	Mostly 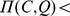 Average	Mostly Broken and Rewired
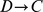	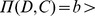 Average	Preserved (this round),
		Broken and Rewired (next round)
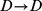	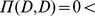 Average	Broken and Rewired
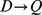	Mostly 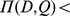 Average	Mostly Broken and Rewired
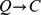	Mostly 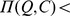 Average	Mostly Broken and Rewired
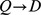	Mostly 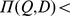 Average	Mostly Broken and Rewired
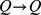	Mostly 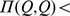 Average	Mostly Broken and Rewired

On the other hand, as the temptation 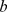 rises, the expected payoffs of agents adopting a quantum strategy 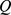 rise at the same time. Particularly, after 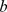 is greater than the critical value, more and more agents using quantum strategies receive payoffs greater than 1, 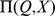 or 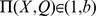. Thereafter, the probability of 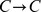 pairs to be broken gradually becomes higher than that of 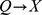 and 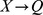 pairs, because cooperators’ payoffs are now often less than the average. Thus, agents using quantum strategies can accumulate higher payoffs, which leads quantum strategies to prevail in the population. Consequently, a quantum strategy becomes the dominant strategy. Note that a defector in a 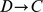 pair always acquires the highest payoff. When 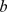 is significantly greater than 1, the defector’s total payoff is thus likely very high. Therefore, the strategy 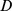 can also be imitated by some myopic agents, but the fraction of defectors rises only a little, because 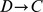 pairs will be broken in the next round, since the opponent (cooperator) minimizes the weight on the 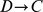 link as punishment in this round.

If the probability of a structural update event, 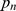, becomes higher, the strategy 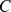 will be dominant in the population in a larger range of 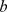. When the time scale for the network evolution is much faster than that for the strategy evolution, say 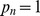, the critical value of 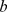 can be increased up to 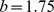. As analyzed above, many 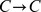 pairs are preserved when 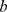 is small. If the structure of the network is updated faster, more 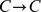 pairs will be preserved and created in terms of the rules of the network evolution. Therefore, the fraction of cooperators in the population is higher at a high probability 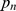 than that at a low probability. When 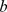 rises further, the number of 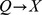 and 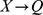 pairs in the population is increased at the same time, but the rate of 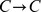 pairs produced is still high, because of the high probability of a structural update event. This slows the spread of quantum strategies, while the strategy 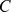 is dominant in the population at a larger critical value of 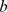.

In summary, the higher the probability of a structural update event, the greater the critical value of 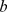 corresponding to the domination of cooperators in the population and the higher the fraction of cooperators. However, in reality, it is often observed that people change their strategies faster than their relationships between friends, i.e., the time scale for the strategy update is faster than that for the structural changes, so in the rest of the paper, the probability of the network evolution 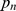 is set at 0.2.

Next, the relationship between the evolution of strategies and the number of neighbors is discussed, and subsequently the impact of punishment on the coevolution of the network structure is investigated. When an initial network is constructed, the number of neighbors of an agent depends on the parameter 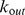, which determines the connectedness of the random network. Therefore, we increase the number of agents’ initial neighbors from 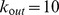 to 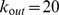 and 30, in order to measure the effects of higher connectedness on the strategy evolution. Comparing them with the result obtained at 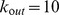 and 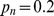, we can see that the critical value of 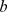 is similar and the fraction of cooperators drops only slightly. It can be inferred that if the number of initial neighbors of agents is equal, i.e., agents have equal chances of success, the results of the coevolution are similar.

**Figure 4:**
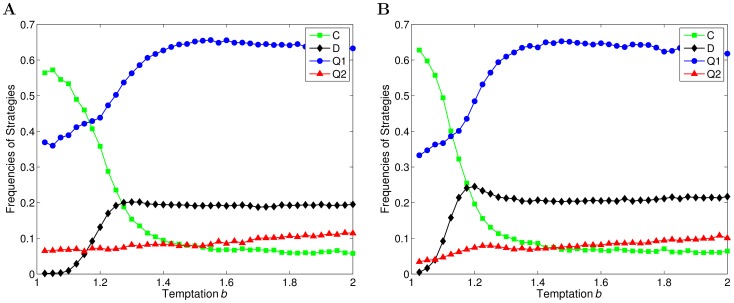
The evolution of strategies as a function of 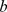 on a weighted and directed evolving network for different intensity of punishment, 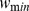. (a) 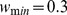. (b) 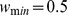. (a)–(b) exhibit the fractions of agents using two quantum strategies (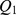 and 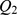) and two classical strategies (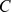 and 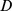) in the population after the coevolution, when 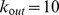, 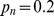 and different intensity of punishment, 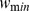, are adopted, respectively.

On the other hand, according to the rules of the network evolution, an agent can break and rewire the links directed from it to the neighbors, if the received payoffs from neighbors are less than the average. On the contrary, if the link between them is directed from the neighbor to the agent, the agent can only minimize the weight on the link in order to punish the neighbor and reduce the neighbor’s total payoff. Thus, the link with a minimal weight 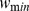 will be broken by the neighbor in the next round, because its payoff is less than the average. In the previous subsection, the minimum of a weight for punishment is 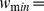
0.1. If the minimum is increased, i.e., the intensity of punishment is reduced, the evolution of strategies and the network will be influenced. Meanwhile, the mean of the normal distribution is increased due to 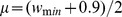. When 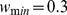 and 0.5, the results depicting the evolution of strategies are shown in Fig. 4.

From Fig. 4, it can be found that with the decrease of the intensity of punishment, the critical value of 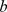 drops significantly from 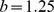 (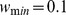) to 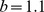 (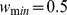). In other words, a quantum strategy is dominant in the population at a smaller 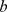, while the fraction of cooperators is reduced at the same time. As is analyzed above, before the critical value of 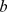, the punishment often occurs among 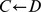 and 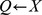 pairs, because the focal agent acquires payoffs less than the average. The punishment causes the agents using quantum strategies cannot accumulate high payoffs and prevents quantum strategies from spreading in the population. However, when 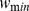 is increased, the intensity of punishment decreases, so that agents adopting quantum strategies can collect high payoffs at a smaller 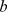. Consequently, a quantum strategy becomes the dominant strategy in the population earlier.

As discussed above, the strategy evolution and the network evolution interact with each other. Finally, we thus investigate the statistical features of the network for different parameters, after the coevolution of strategies and the network structure comes to an end. The clustering coefficient and the average path length are most often used to describe statistical features of network topology. Hence, we calculate these quantities before and after the coevolution as representative measures of the network structure. The clustering coefficient is a measure of degree to which nodes in a network tend to cluster together. In this paper, the local clustering coefficient for a directed network is used, which is given as
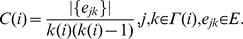
(10)


Here, 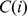 is the clustering coefficient of the node 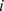, while the clustering coefficient of the network is the average of clustering coefficients of all nodes, 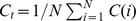. The average path length is the average of the shortest paths for all pairs of nodes in a network, which has the form
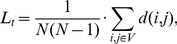
(11)where 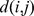 denotes the shortest distance between nodes 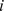 and 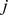.

Initially, the evolution starts on a regular random graph, whose clustering coefficient 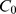 and average path length 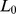 both are small. After the model is iterated for 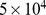 times, the structure of the network is changed largely, so the clustering coefficient 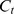 and average path length 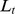 of the network are calculated again, which are listed in Table 2. The clustering coefficient 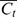 and average path length 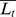 in Table 2 corresponding to different 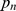 and 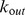 are the averages of many 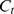 and 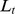 that are obtained from different independent initial conditions, respectively. From Table 2, it can be found that the clustering coefficient 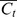 rises considerably compared to 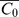. It is even 20 times greater than 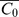 in the case of 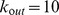. On the contrary, with the increase of 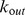, the growth rate of 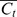 drops, but it is still greater than 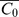. If the probability of a structural update event rises from 0.2 to 1, while 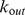 is a constant, the clustering coefficient 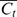 will rise slightly with it. On the other hand, the average path length 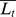, after the coevolution, is not very different from the initial average path length 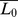. By further observation, it can be seen that the average path length 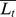 is similar, when the probability of a structural update event rises, whereas it decreases with the increase of 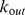. Summing up, after the network evolves according to the rules of the network evolution, a large clustering coefficient and small average path length emerge in the network, which are properties that are frequently referred to as small-world properties. This is because in our coevolutionary model, the rules concerning the evolution of the network structure allow agents to break the links that belong to them, and then to rewire these links to neighbors’ neighbors at random. This rewiring operation, while keeping the average path length small, increases the number of links among agents’ neighbors, which is the main reason for the emergence of the relatively large (compared to that of a random network) clustering coefficient. In addition, the large clustering coefficient can also be interpreted as emerging because of the tendency of each agent to organize and sustain cohesive clusters of reciprocal trust.

**Table 2:** Comparison of statistical features of networks before and after coevolution.

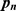	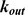	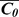	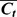	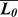	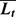
0.2	10	0.0030	0.0637	3.5861	3.7422
0.5	10	0.0030	0.0642	3.5826	3.7485
1.0	10	0.0031	0.0658	3.5833	3.7468
0.2	20	0.0071	0.0392	2.9725	2.9712
0.2	30	0.0111	0.0346	2.6936	2.7006

**Figure 5:**
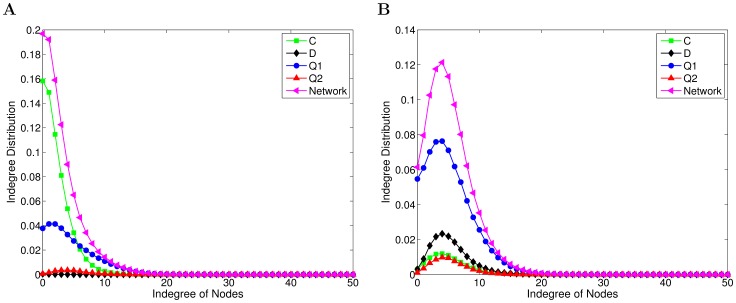
The indegree distributions of networks. (a) 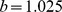. (b) 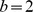. Both panels (a) and (b) show the indegree distributions separately for different strategies as well as for the network as a whole, as depicted in the legends. In both cases we have used 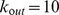, 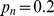 and 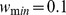, and the results are averages over 200 different initial conditions.

Lastly, we also investigate the degree distribution of networks, which is also an important statistical feature. In this paper, we focus on the indegree distributions of directed networks because the outdegree is fixed, which is defined to be the fraction of nodes in the network with indegree 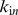, namely, 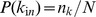, where 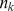 is the number of nodes with indegree 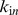. Further, among the nodes with the same strategy in the network, we study the indegree distribution under a strategy in order to find possible correlations between the indegree distributions and strategies. The indegree distribution under a strategy is defined as 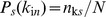, where 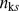 represents the number of nodes with indegree 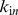 and where these nodes use the same strategy. Figure 5 shows the indegree distributions at different 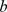, which are the statistical averages over 200 independent realizations with different initial conditions.

Comparing the indegree distributions of networks 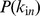 at different 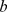, we can see that the indegrees of most nodes (19%) in networks are zero or one at 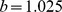, while at 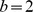, the indegrees of 12% of nodes are five. Further, observing the indegree distributions under different strategies 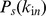, it can be found that when 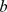 is small, nodes with larger indegrees are those using strategies 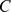 and 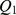. On the contrary, at 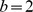, they are those with strategies 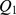 and 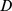, and the nodes with the largest indegrees are quantum strategists. As we have analyzed above, the strategy 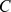 and the strategy 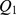 dominate in the population at 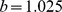 and 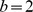 respectively, which indicates that most nodes in the network adopt 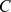 at 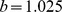 or 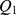 at 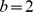. During the network evolution, nodes with these strategies thus have a higher chance to be connected by other nodes, which directly leads to the fact that these are also the nodes with the largest indegees, as can be inferred from Fig. 5.

## Conclusions

We have proposed and studied a model with coevolutionary rules, which uses an evolving network to represent the relationships among agents. Based on the model, the evolution of quantum and classical strategies on an evolving network is investigated. The coevolution starts on a regular random network, in which the number of each agent’s neighbors is equal and the weights on links are one. The same number of neighbors guarantees each agent has the same ability to “make friends”, while the direction of a link indicates to whom the link belongs, but agents can adopt strategies from one another likewise unrestricted by the directionality of links.

If strategies evolve on a static network, a quantum strategy becomes the dominant strategy in the population from the outset. However, when the network evolution is involved, even if the probability of a structural update event 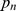 is low, cooperators are dominant in the population instead of agents using quantum strategies when 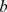 is small. As the probability 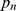 rises, cooperators prevail in a larger range of 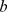. But, finally, a quantum strategy defeats the classical strategies and becomes the dominant strategy in the population. When the probability of the network evolution remains constant, similar results of the coevolution are obtained, even if initially the number of neighbors of each agent is increased. On the other hand, if the intensity of punishment is reduced by increasing 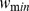, a quantum strategy can dominate in the population at a smaller 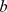.

After the coevolution ends, the structure of the network is changed largely due to links being broken and rewired. By analyzing the statistical features of the network before and after the coevolution, we can find that the average path length increases slightly, but the clustering coefficient increases significantly after the coevolution, in particular it increases about 20 times at 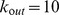 compared to that before the evolution. The growth rate of the clustering coefficient decreases with the increase of 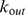. It can be concluded that small world properties, small average path length and a large clustering coefficient, emerge spontaneously in the network after the coevolution. Comparing the indegree distributions of networks under different strategies 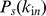, it can be found that at different 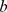, nodes with the larger indegrees are cooperators and quantum strategists, respectively.
